# Anti-Atopic Properties of Gracillin Isolated from *Dioscorea quinqueloba* on 2,4-Dinitrochlorobenzene-Induced Skin Lesions in Mice

**DOI:** 10.3390/nu10091205

**Published:** 2018-09-01

**Authors:** Jonghwan Jegal, No-June Park, Beom-Geun Jo, Sim-Kyu Bong, Hyun Jegal, Min Hye Yang, Su-Nam Kim

**Affiliations:** 1 College of Pharmacy, Pusan National University, Busan 46241, Korea; jhjegal@pusan.ac.kr (J.J.); dtc98103@pusan.ac.kr (B.-G.J.); 2 Natural Products Research Institute, Korea Institute of Science and Technology, Gangneung 25451, Korea; parknojune@kist.re.kr (N.-J.P.); 115044@kist.re.kr (S.-K.B.); 116524@kist.re.kr (H.J.)

**Keywords:** gracillin, *Dioscorea quinqueloba*, 2,4-dinitrochlorobenzene, atopic dermatitis, skin barrier recovery, interleukin 4

## Abstract

Naturally occurring saponins have been reported to have anti-inflammatory and immunomodulatory effects. However, the effects of gracillin, a main saponin component of *Dioscorea quinqueloba* (*D. quinqueloba*), on atopic dermatitis (AD), have not been previously studied. The aim of this study was to determine whether gracillin isolated from *D. quinqueloba* has an anti-AD effect on 2,4-dinitrochlorobenzene (DNCB)-induced AD-like skin lesions in SKH-1 hairless mice. Topical co-treatment of gracillin and DNCB for two weeks markedly reduced symptoms typical of AD (redness, itching, swelling and skin lichenification), decreased transepidermal water loss (TEWL) and increased skin hydration. In addition, gracillin strongly inhibited PI-induced IL-4 expression in RBL-2H3 cells and in the skins of AD mice. Our results suggest gracillin is a potential candidate for the prevention and treatment of AD and other inflammatory skin disorders.

## 1. Introduction

Atopic dermatitis (AD) is one of the most common inflammatory skin diseases [1] and is characterized by severe itching, xeroderma, eczematous and erythematous plaques with oozing exudates [2]. The lifetime prevalence of AD is about 3–10% in adults and up to 20% in children [1,3], and is more common in industrialized than rural areas [3]. Atopic diseases are classified as extrinsic or intrinsic. Extrinsic AD has a higher prevalence [4] and is a hypersensitive or allergic response mediated by immunoglobulin E (IgE) [5]. Extrinsic AD is triggered by a variety of allergic factors that include food, pollen and house dust mites [5]. Elevations in serum interleukin-4 (IL-4; an inflammatory cytokine produced by TH2 cells) levels and IL-4 receptor mRNA expression are the most distinctive manifestations of extrinsic AD [6], and treatment with calcineurin inhibitors, such as tacrolimus and pimecrolimus, relieve atopic skin symptoms by inhibiting IL-4 expression [7].

Saponins are steroid or triterpenoid glycosides that are present in most plants like legumes and yams [8,9] and much research has been focused on the extraction, isolation and purification of saponins from diverse natural products due to their varied physicochemical properties and biological activities [9]. Saponins have cytotoxic, immunostimulant, antiphlogistic, anti-tumor, antimicrobial, antifungal, antioxidant and cholesterol lowering properties [9,10,11]. The anti-catarrhal and membrane-disrupting properties of saponins have led to their use for the treatment of respiratory ailments, such as nasal and sinus congestion, mucosal edema and productive cough [12]. According to recent studies, the anti-allergic and anti-inflammatory properties of plant-derived saponins strongly support their use as potential anti-atopic agents [13,14].

The genus *Dioscorea* (commonly referred to as ‘yams’) is composed of around 650 species. The yam (*Dioscorea* spp.) is widely cultivated in Africa, Asia and Central and South America and is a rich source of starch [11,15]. In East Asia, dried rhizomes of yam have been used as a traditional folk medicine for asthma, rheumatoid arthritis and bronchitis [16].

*Dioscorea* species contain diverse health promoting substances, such as amylose, cholin, mucin, steroidal saponins and sapogenins [17]. Although a number of studies have been conducted on various species of the *Dioscorea* genus, few have addressed the subject in *D. quinqueloba*, and the anti-atopic activities of saponins isolated from *D. quinqueloba* have not been previously examined. In a previous study, we found that an extract of the rhizomes of *D. quinqueloba* exhibited meaningful therapeutic effects against AD in oxazolone- and 2,4-dinitrochlorobenzene (DNCB)-induced AD murine models [18]. The present study was undertaken to isolate gracillin, the major phytochemical in *D. quinqueloba,* and to determine its anti-atopic effects in a murine model of AD. The results show that 1% gracillin has an anti-AD effect comparable to that of 1% *D. quinqueloba* extract as demonstrated by the lack of increased atopic skin symptoms after sensitization of DNCB.

## 2. Materials and Methods

### 2.1. Spectroscopy and Column Chromatography

^1^H and ^13^C NMR (Nuclear Magnetic Resonance), COSY (Correlation Spectroscopy), HMQC (Heteronuclear Multiple-Quantum Correlation Spectroscopy), HMBC (Heteronuclear Multiple-Bond Correlation Spectroscopy), and NOESY (Nuclear Overhauser Effect Spectroscopy) spectral data were obtained using an Agilent Superconducting FT-NMR 400–500 MHz Spectrometer (Agilent Technologies, CA, USA) (Supplementary Figures S1 and S2). HR-ESI mass spectra were recorded on a 6530 Accurate-Mass Q-TOF LC/MS (Agilent Technologies, CA, USA). Column chromatography was performed using silica gel (230–400 mesh; Merck, Darmstadt, Germany).

### 2.2. Plant Material and Isolation of Gracillin from D. qinqueloba

**Figure 1:**
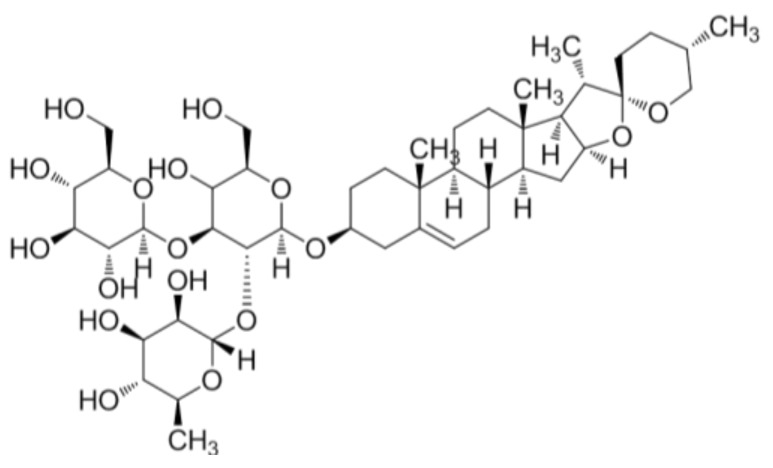
Chemical structure of gracillin isolated from *Dioscorea quinqueloba* rhizomes.

Rhizomes of *D. quinqueloba* were acquired from Jirisan Hanbang Foods (Sancheong, Gyeongnam, South Korea) and verified by Professor Eun Ju Jeong of the Department of Agronomy and Medicinal Plant Resources, Gyeongnam National University of Science and Technology. A voucher specimen (PNU-0023) was deposited at the Medicinal Herb Garden, Pusan National University. Dried chopped rhizomes (20 kg) were extracted with 95% EtOH and evaporated under reduced pressure to yield *D. quinqueloba* EtOH extract (670 g). The obtained EtOH extract was then suspended in distilled water and partitioned with *n*-BuOH. The obtained *n*-BuOH fraction (300 g) was subjected to silica gel column chromatography using a CH_2_Cl_2_–MeOH gradient system (10:1 → 100% MeOH) as eluent and yielded 14 fractions (DQB1–DQB14). Gracillin (2.637 g) was obtained by recrystallization of DQB8 from MeOH (Figure 1).

### 2.3. RBL-2H3 Cell Culture

The RBL-2H3 cell line (a rat basophilic leukemia cell line) was purchased from the American Type Culture Collection (CRL-2256, Bethesda, MD, USA). Cells were cultured in minimum essential medium (MEM) supplemented with Eagle’s salt containing 10% fetal bovine serum (FBS), 2 mM l-glutamine, 100 U/mL penicillin and 100 μg/mL streptomycin at 37 °C in a humidified 5% CO_2_/95% air atmosphere.

### 2.4. Release of IL-4 from RBL-2H3 Cells

RBL-2H3 cells were seeded in MEM/10% FBS containing dimethyl sulfoxide (DMSO) or gracillin (10 μM) for 30 min and then treated with phorbol 12-myristate 13-acetate/ionomycin (PI) for 16 h to induce an AD-like condition. The cells were then harvested to synthesize cDNA and quantitative real-time PCR (qPCR) was used to measure IL-4 mRNA expressions. Total RNA was isolated from cells using RNAiso Reagent (TaKaRa, Shiha, Japan) according to the manufacturer’s instructions. PCR product accumulations were observed directly by checking increases in the reporter dye (SYBR). At each time point, cytokine expression levels in treated cells were compared to those in controls using the comparative cycle threshold (Ct) method. The sequences of the primers used were: IL-4 forward: 5′-ACC TTG CTG TCA CCC TGT TC-3′; IL-4 reverse: 5′-TTG TGA GCG TGG ACTCAT TC-3′; β-actin forward: 5′-TCA TCA CCA TCG GCA ACG-3′, β-actin reverse: 5′-TTC CT GAT GTC CAC GTC GC-3′. Transcribed product amounts were calculated after normalization with respect to β-actin.

### 2.5. β-Hexominidase Secretion Assay

RBL-2H3 cells were sensitized overnight with DNP-specific dinitrophenyl immunoglobulin E (IgE), washed with Siraganian buffer, exposed to DMSO or gracillin (10 μM) for 1 h and stimulated with DNP-BSA antigen (1 μg/mL) to induce degranulation. The supernatants were transferred into 96-well plates and incubated with 1 mM of 4-nitrophenyl-*N*-acetyl-β-d-glucosaminide as substrate in 0.1 M citrate buffer for 3 h at 37 °C. Absorbance was measured using a microplate reader at 405 nm.

### 2.6. Animals

Six-week-old female SKH-1 hairless mice were purchased from Orient Bio Inc. (Seongnam, Republic of Korea) and housed in a ventilated, controlled room (25 ± 5 °C, 55 ± 5% RH (relative humidity)) with free access to water and standard laboratory food. All animal experiments were performed in accordance with the Guide for the Care and Use of Laboratory Animal of the National Institutes of Health (NIH publication No. 85-23, revised 2011) after obtaining permission from the Institutional Animal Care and Use Committee of KIST (Certification no. KIST-2016-011).

### 2.7. Skin Symptoms after Treatment with Gracillin

To induce AD in SKH-1 hairless mice, 2,4-dinitrochlorobenzene (DNCB) (Sigma-Aldrich, Seoul, South Korea) was used. DNCB (1%; 100 μL in propylene glycol:EtOH = 7:3) was spread onto dorsal skin daily for 7 days. After this sensitization period, mice were challenged with DNCB (0.1%; 100 μL) every 3 days for an additional 2 weeks (the DNCB group). Alternatively, sensitized mice were treated with gracillin twice a day for 2 weeks and with DNCB as described above (the DNCB-gracillin group). When DNCB and gracillin were administered on the same days, gracillin was administered 4 h before DNCB. Vehicle controls were treated with propylene glycol/EtOH solution (the CON group) as described for DNCB above.

### 2.8. Histological Examination

To evaluate histopathological changes, the dorsal skins of SKH-1 hairless mice were fixed in 10% formalin for 24 h, embedded in paraffin, sectioned at 2–3 mm, transferred to slides, dried overnight at 37 °C and dyed with hematoxylin and eosin (H&E) or toluidine blue. Histopathological changes were examined using an optical microscope (Olympus CX31/BX51, Olympus Optical Co., Tokyo, Japan) and photographed (TE-2000U, Nikon Instruments Inc., Melville, LA, USA). The thickness of the epidermis was measured using a ruler equipped with a microscope and the LAS v4.8 (Leica Microsystem, Herbrugg, Switzerland) program.

### 2.9. Measurement of Transepidermal Water Loss (TEWL) and Skin Hydration

Transepidermal water loss (TEWL) and skin hydration were measured to evaluate skin barrier repair using a Tewameter TM210 (Courage and Khazaka, Cologne, Germany) and a SKIN-O-MAT (Cosmomed, Ruhr, Germany). These measurements were made weekly under controlled conditions (25 ± 5 °C, 55 ± 5% RH).

### 2.10. Measurement of Total Serum IgE and IL-4 Levels

Blood samples were collected and centrifuged at 10,000 rpm for 15 min at 4 °C. Serum samples were stored at −70 °C until required for total IgE and IL-4 determinations, which were performed using enzyme-linked immunosorbent assay (ELISA) kits (eBioscience, San Diego, CA, USA).

### 2.11. Statistical Analysis

The analysis was conducted using one-way analysis of variance (ANOVA) and a statistical software program. Results are presented as means ± SDs (Standard Deviations) and statistical significance was accepted for *p* values < 0.05.

## 3. Results

### 3.1. Effects of Gracillin on IL-4 Expression and β-hexosaminidase Release in RBL-2H3 Cells

**Figure 2:**
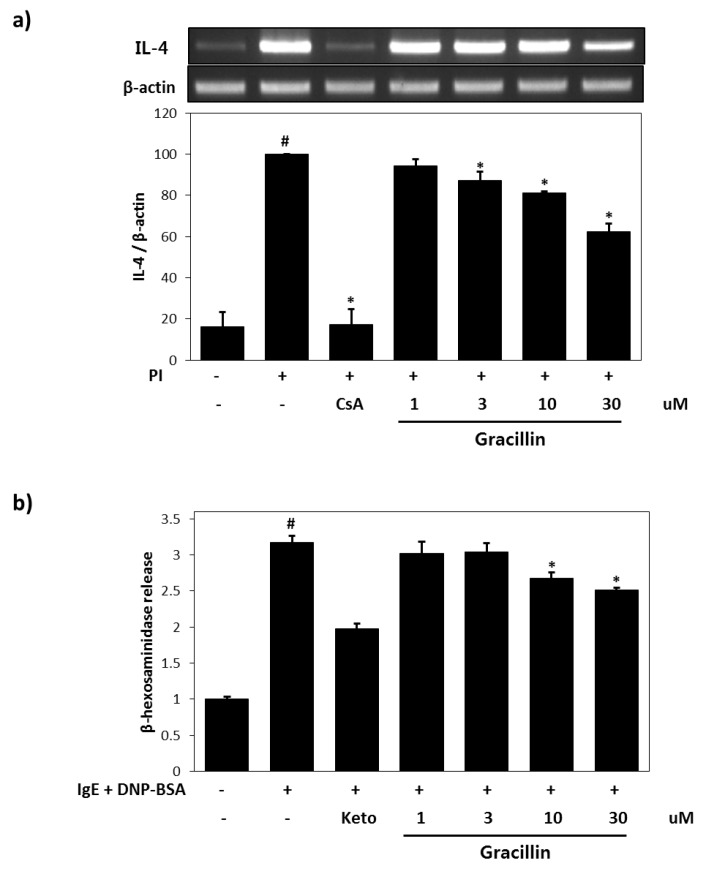
Anti-inflammatory effects of gracillin in rat basophilic leukemia cell line (RBL-2H3) cells. (**a**) Effects of gracillin on interleukin 4 (IL-4) mRNA expression in PMA/ionomycin (PI)-mediated RBL-2H3 cells. Results are expressed as means ± SDs (Standard Deviations) of two independent experiments. (**b**) Effects of gracillin on β-hexosaminidase release from immunoglobulin E (IgE)-mediated RBL-2H3 cells. Results are expressed as the means ± SDs of two independent experiments. ^#^ *p* < 0.05 vs. vehicle control; * *p* < 0.05 vs. PI. CsA: 1 μM Cyclosporin A, Ket: 35 μM Ketotifene.

The suppressive effect of gracillin on IL-4 expression was investigated using PI-stimulated RBL-2H3 cells. IL-4 mRNA expression was significantly increased by PI treatment for 16 h (6.7-fold versus vehicle controls) (Figure 2a). However, IL-4 mRNA expression was lower in cells pre-treated with gracillin (30 μM) and then stimulated with PI, than in PI treated controls. DNP has been previously shown to increase β-hexosaminidase release in IgE-sensitized RBL-2H3 cells [19]. We found that gracillin markedly inhibited β-hexosaminidase release in RBL-2H3 cells. DNP-specific IgE alone caused significant β-hexosaminidase release (3.2-fold versus non-treated controls), but treatment with 30 µM gracillin reduced this increase by 31.8% (Figure 2b).

### 3.2. Effects of Gracillin on AD-like Skin Symptoms Induced by DNCB

**Figure 3:**
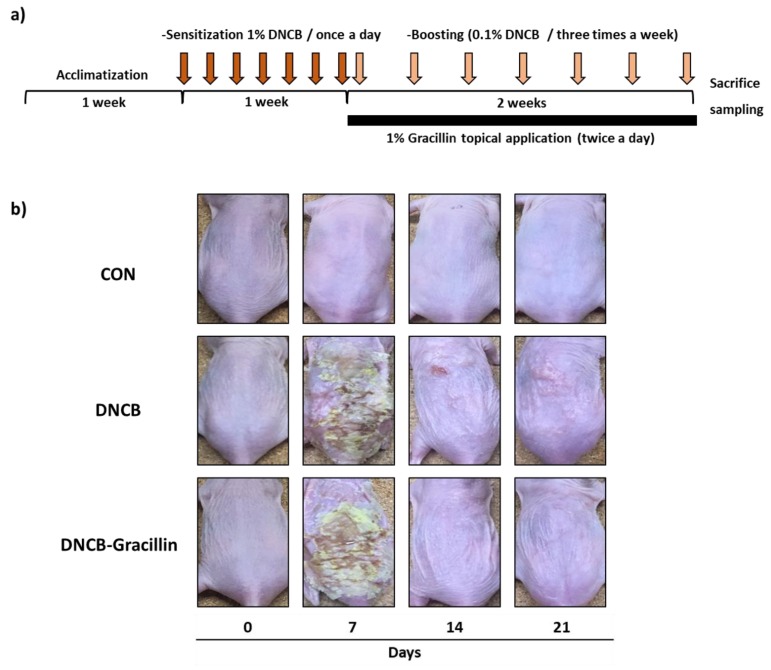
Effects of gracillin on pathological changes in the skins of 2,4-dinitrochlorobenzene (DNCB)-sensitized atopic hairless mice. (**a**) Schematic representation of the experiment. (**b**) Clinical features of atopic dermatitis (AD)-like dorsal skin lesions. CON: vehicle controls, DNCB: DNCB controls, DNCB-Gracillin: DNCB plus 1% gracillin treated mice.

**Figure 4:**
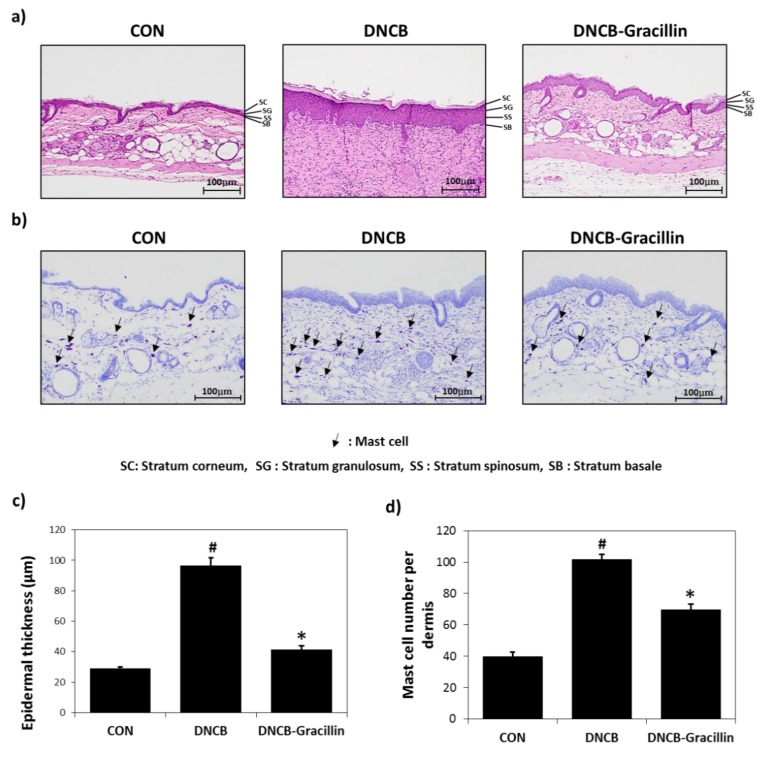
Histopathological effects of gracillin in DNCB-sensitized atopic hairless mice. (**a**) Histopathological features of dorsal skin lesions in 1% gracillin plus DNCB co-treated mice as determined by hematoxylin and eosin (H&E) staining. (**b**) Histopathological features of dorsal skin lesions in 1% gracillin plus DNCB co-treated mice as determined by toluidine blue staining. Tissues were excised, fixed in 10% formaldehyde, embedded in paraffin, sectioned and stained with H&E (magnification, 100×) or toluidine blue. (**c**) Epidermal thicknesses. (**d**) Mast cell densities in dermis. Results are presented as means ± SDs (*n* = 7). The means ± SEMs of two independent experiments performed in triplicate are shown. ^#^ *p* < 0.05 vs. vehicle controls; * *p* < 0.05 vs. DNCB treated controls.

As shown in Figure 3b, erythema, dry skin, welling, parakeratosis, exudation, and excoriation were observed in the dorsal skins of SKH-1 hairless mice after treatment with DNCB, and these lesions were significantly improved by the topical application of gracillin for 2 weeks. To investigate histologic changes, tissue samples obtained from the mice were stained with H&E or toluidine blue. H&E staining showed gracillin markedly alleviated epidermal hypertrophy in DNCB-treated AD mice (Figure 4a). In addition, H&E and toluidine blue staining results demonstrated that the number of mast cells in lesioned skins was significantly reduced by gracillin (Figure 4a,b). Treatment with DNCB for 21 days caused a significant increase in dermal thickness of 3.4-fold versus the control, and gracillin inhibited this DNCB-induced increase in skin thickness by 85.7% (Figure 4c). DNCB also increased in the number of mast cells in the dermis by 2.5-fold versus the control, and gracillin reduced DNCB-induced mast cell infiltration by 50% (Figure 4d).

### 3.3. Effects of Gracillin on Skin Barrier Function

**Figure 5:**
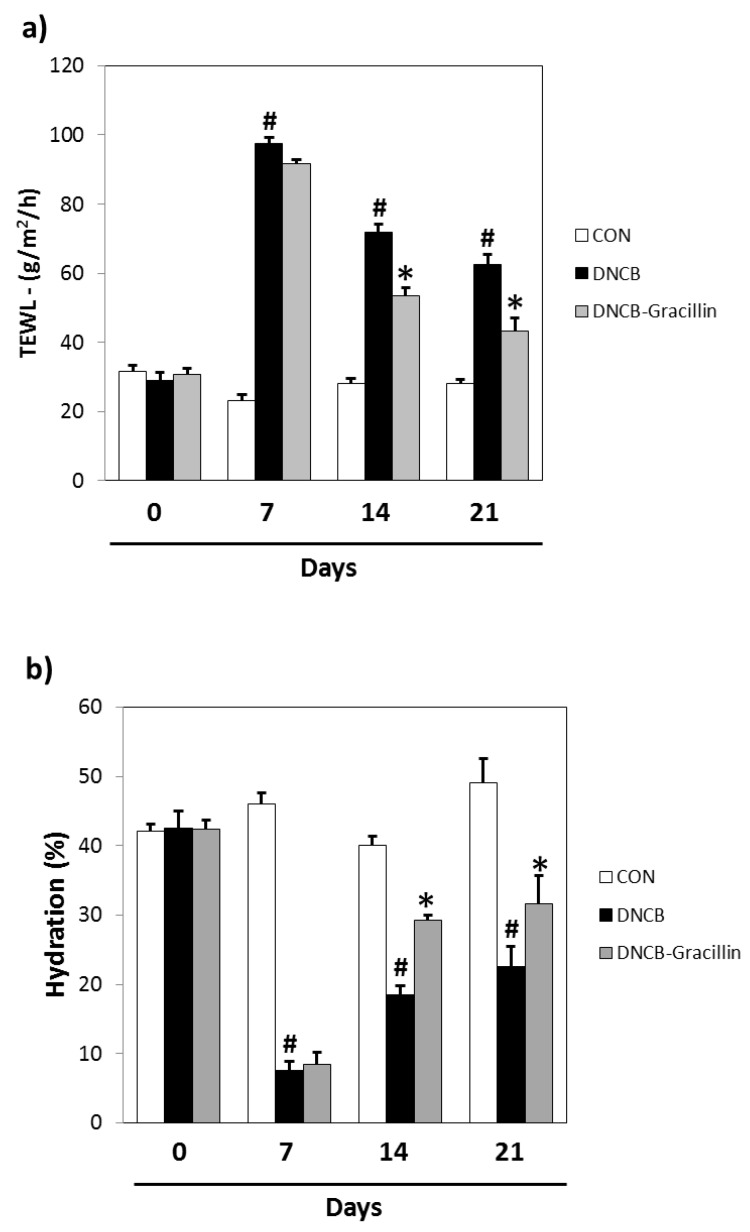
Effects of gracillin on skin barrier function in DNCB-sensitized atopic hairless mice. (**a**) Transepidermal water loss (TEWL) values. (**b**) Skin hydration values. CON: vehicle controls, DNCB: DNCB-controls, DNCB-Gracillin: DNCB plus 1% gracillin treated mice. Results are presented as the means ± SDs (*n* = 7) of two independent experiments performed in triplicate. ^#^ *p* < 0.05 vs. vehicle controls; * *p* < 0.05 vs. DNCB controls.

Topical DNCB reduces skin barrier function as determined by TEWL and skin hydration testing. In DNCB-treated animals, TEWL increased from 23.2 to 97.6 g/m^2^/h and skin hydration decreased from 46.0 to 7.6% versus vehicle controls. After 21 days of treatment, significant differences were observed between the gracillin-DNCB and DNCB groups. Co-treatment with 1% gracillin reduced the decrease in TEWL observed in the DNCB control by more than half (Figure 5a) and increased skin hydration by 39.8% (Figure 5b).

### 3.4. Effects of Gracillin on Serum IgE and IL-4 Levels in DNCB-Induced Atopic Mice

**Figure 6:**
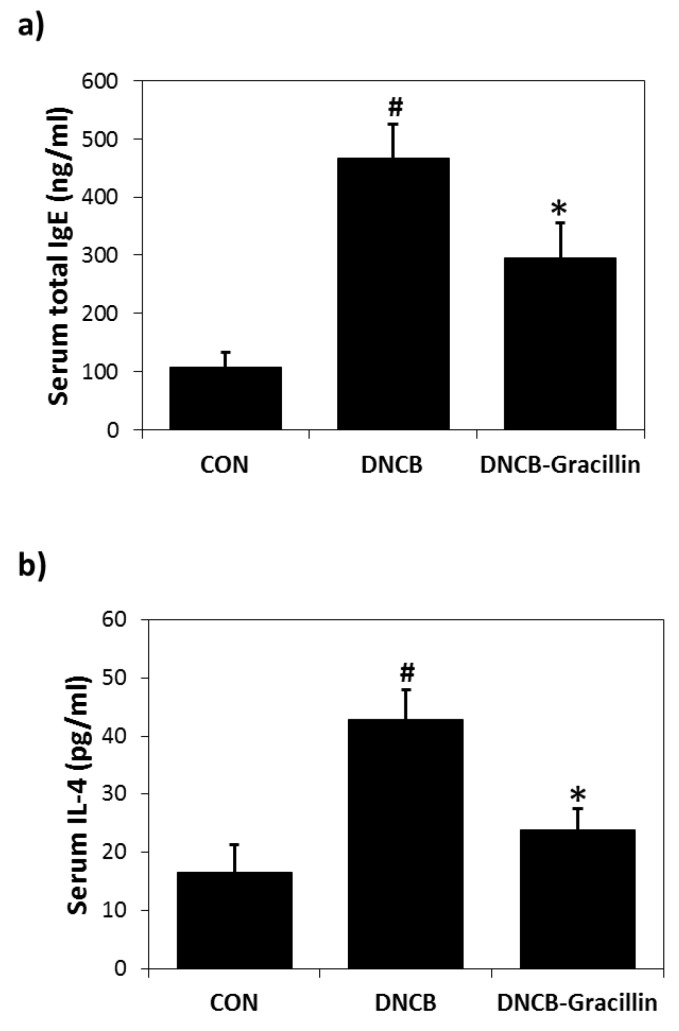
Effects of gracillin on serum IgE and IL-4 levels in DNCB-induced atopic hairless mice. (**a**) Total serum IgE levels. (**b**) Total serum IL-4 levels. Results are presented as the means ± standard errors (*n* = 7) of two independent experiments performed in triplicate. ^#^ *p* < 0.05 vs. vehicle controls; * *p* < 0.05 vs. DNCB controls.

DNCB-induced skin inflammation was accompanied by increases in serum IgE and IL-4 levels, and gracillin co-treatment inhibited these DNCB-induced increases by 48.6% (Figure 6a) and 71.4% (Figure 6b), respectively.

## 4. Discussion

Atopic dermatitis is one of the most common allergic diseases and is characterized by pruritic eczematous skin lesions [3]. Although AD is a multifactorial disease, skin barrier dysfunction is considered to be an important initiator in the development of AD [20]. Defective skin barrier function can increase skin susceptibility to various irritants and allergens, and thus contributes to the pathogenesis of AD [20]. For this reason, topical emollients and moisturizers that increase skin hydration are often used by AD patients [7], and natural products are commonly used to moisturize dry or sensitive skin [21]. Of the several types of phytochemicals, saponins have been found to possess anti-inflammatory, anti-allergic, immunoregulatory and skin moisturizing properties [10]. Accordingly, saponins derived from natural sources are considered to be worth studying as potential AD medicines.

In a previous study, we found that a *D. quinqueloba* extract (DQ) potently inhibited IL-4 expression in RBL-2H3 cells [18]. Subsequent in vivo experiments undertaken to evaluate the anti-AD effects of DQ on oxazolone or DNCB-induced dermatitis in mice showed that both topical and broad application of DQ alleviated atopic skin symptoms. Based on these results, we isolated and characterized gracillin, a major saponin component of DQ, and studied its anti-inflammatory and anti-atopic activities. In vitro studies showed gracillin had concentration-dependent inhibitory effects on IL-4 expression and degranulation in RBL-2H3 cells. Furthermore, in DNCB-sensitized hairless mice, gracillin attenuated both IL-4 overexpression and IgE hyperproduction. IL-4 is one of two cytokines that induce IgE synthesis, and elevated IL-4 and IgE levels are known to be closely associated with T_H_2-type allergic inflammatory responses in AD patients [22,23]. Thus, we considered that treatment with gracillin might prevent the initiation of AD-like skin lesions by inactivating T_H_2-type cells.

Topical corticosteroids have been used as the first line therapy for mild to severe AD for 50 years [7,24]. Although the therapeutic effects of topical corticosteroids on AD have been proven, they also have the potential to cause diverse side effects, such as skin thinning, striae distensae, petechiae, telangiectasia, and acne [7]. Not unexpectedly, these adverse effects cause patients to be cautious of steroid use, which reduces patient compliance and treatment effectiveness [25]. Steroid-free calcineurin inhibitors, such as tacrolimus and pimecrolimus, are a new class of topical preparations that were specifically developed to treat AD [26]. Tacrolimus and pimecrolimus act as immunomodulators and anti-inflammatory agents and markedly inhibit T lymphocyte activation [26,27], but they are expensive and not effective in every case [27]. According to our findings, gracillin relieves atopic skin symptoms and inhibits IL-4 overexpression in DNCB-induced AD mice, which suggests it is a potential effective candidate for AD therapy in terms of cost and efficacy.

Atopic dermatitis is a disease associated with reduced epidermal barrier function and immune dysregulation [28,29]. Impairments in skin barrier function facilitate allergen penetration and promote allergic contact sensitization [30]. Moisturizers and emollients enhance skin barrier function, and thereby are effective at preventing AD [31]. In fact, several clinical studies have shown long-term treatment with moisturizers ameliorates AD and reduces TEWL [32,33]. In the present study, changes in TEWL and skin hydration confirmed that gracillin triggers skin barrier function recovery of atopic dry skin, which suggests gracillin might be used to treat atopic dry skin.

## 5. Conclusions

In conclusion, topical application of gracillin, a primary saponin from *D. quinqueloba* rhizomes, was found to effectively reduce atopic skin symptoms, such as itching, redness, and skin thickening, in our DNCB-induced AD murine model. Impaired skin barrier function and reduced skin hydration were significantly improved in AD mice treated with gracillin for two weeks, and gracillin also inhibited IL-4 overproduction in vivo and in vitro. Further clinical studies are warranted to confirm the anti-atopic effects of gracillin on human skin.

## Supplementary Materials

The following are available online at http://www.mdpi.com/2072-6643/10/9/1205/s1, Figure S1: The ^1^H NMR spectrum of gracillin (400 MHz, Py-d5), Figure S2: The ^13^C NMR spectrum of gracillin (150 MHz, Py-d5).
