# Supplementary material for: Anti-Atopic Properties of Gracillin Isolated from Dioscorea quinqueloba on 2,4-Dinitrochlorobenzene-Induced Skin Lesions in Mice
Source: Nutrients. 2018 Sep 1;10(9):1205. doi: 10.3390/nu10091205 (PMC6164938; doi:10.3390/nu10091205)

SUPPLEMENTARY DATA

Anti-Atopic Properties of Gracillin Isolated from *Dioscorea quinqueloba* on 2,4-Dinitrochlorobenzene-Induced Skin Lesions in Mice

Jonghwan Jegal[1,†], No-June Park[2,†], Beom-Geun Jo[1], Sim-Kyu Bong[2], Hyun Jegal[2], Min Hye Yang[1,*] and Su-Nam Kim[2,*]

Affiliation

[1] College of Pharmacy, Pusan National University, Busan 46241, South Korea; jhjegal@pusan.ac.kr (J.J.); dtc98103@pusan.ac.kr (B.-G.J.)

[2] Natural Products Research Institute, Korea Institute of Science and Technology, Gangneung 25451, South Korea; 115519@kist.re.kr (N.-J.P.); 115044@kist.re.kr (S.-K.B.); 116524@kist.re.kr (H.J.)

* Correspondence: mhyang@pusan.ac.kr (M.H.Y.); snkim@kist.re.kr (S.-N.K.);

Tel.: +82-51-510-2811 (M.H.Y.); +82-33-650-3503 (S.-N.K.);

Fax: +82-51-513-6754 (M.H.Y.); +82-33-650-3419 (S.-N.K.)

Supporting Information Contents:

Figure S1. The ^{1}H NMR spectrum of gracillin (400 MHz, Py-d_5).

Figure S2. The ^{13}C NMR spectrum of gracillin (150 MHz, Py-d_5).

Figure S1. The ^{1}H NMR spectrum of gracillin.

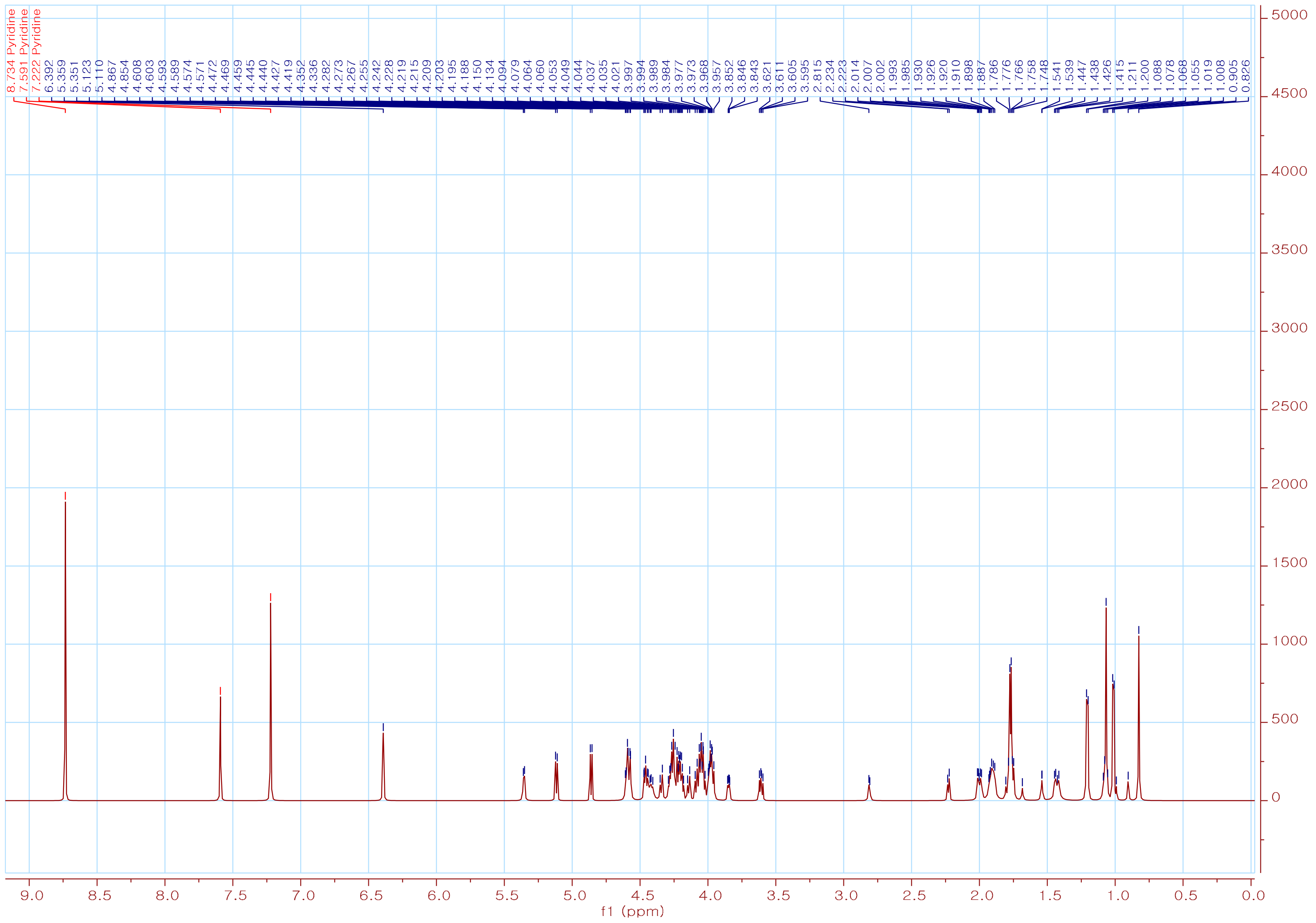

Figure S2. The ^{13}C NMR spectrum of gracillin.

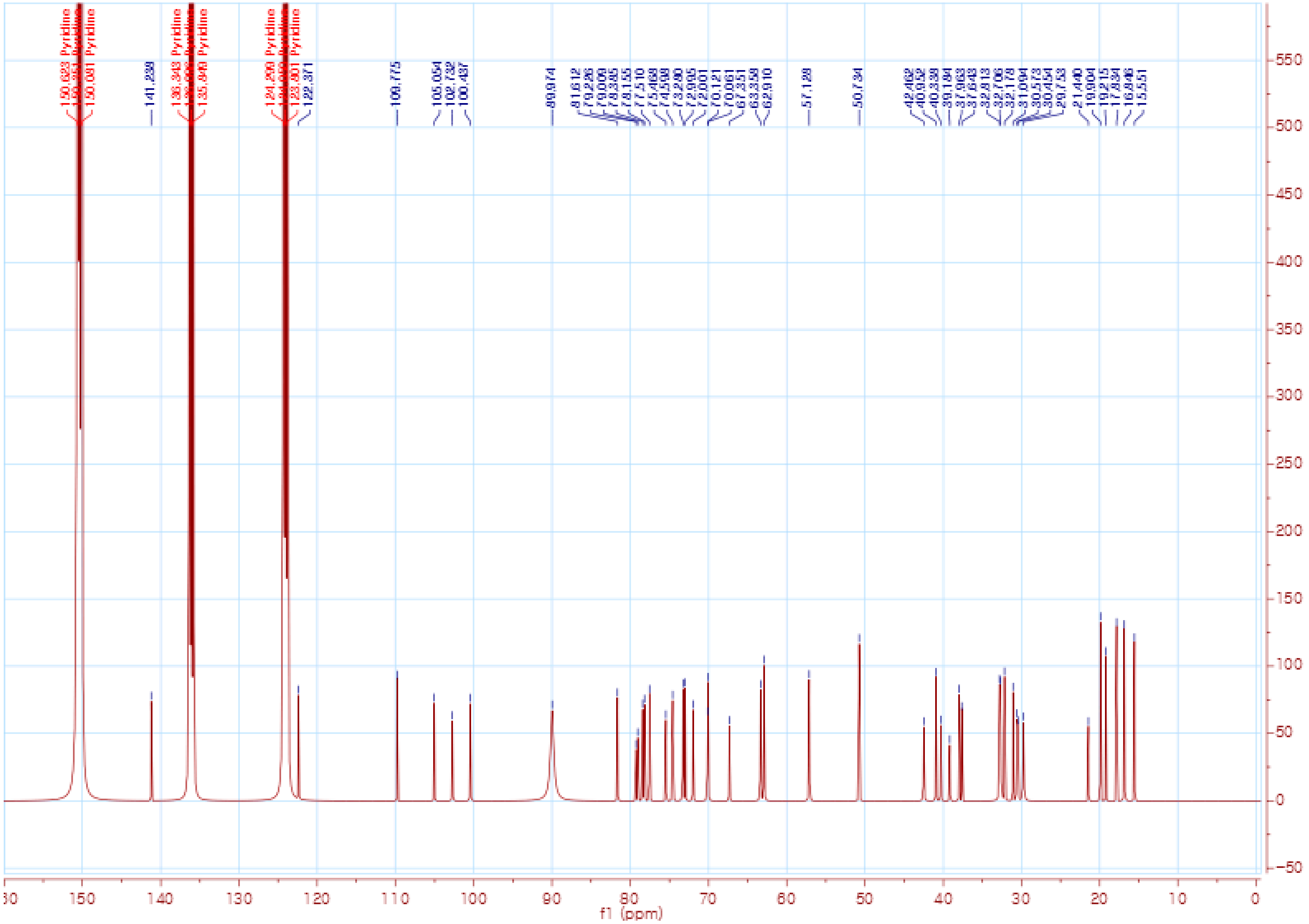

Supplement: Supplementary file 1 [file nutrients-10-01205-s001.pdf]
